# Clinicohistological correlation of etiological spectrum of chronic liver disease diagnosed during noncirrhotic stages in children: Can need of liver biopsy be obviated?

**DOI:** 10.1002/jgh3.12441

**Published:** 2020-10-30

**Authors:** Tryambak Samanta, Rajarshi Basu, Radheshyam Purkait, Sudipta Kar, Debasis Das, Sutapa Ganguly

**Affiliations:** ^1^ Department of Medical Gastroenterology Medical College Kolkata India; ^2^ Department of Pediatrics Nil Ratan Sircar Medical College Kolkata India; ^3^ Department of Community Medicine Medical College Kolkata India; ^4^ Department of Pediatrics KPC Medical College Kolkata India

**Keywords:** children, chronic liver disease, clinicohistological correlation, liver biopsy, noncirrhotic stage

## Abstract

**Background and Aim:**

Limited data exist regarding the etiological spectrum of the subset of chronic liver diseases (CLDs) diagnosed in noncirrhotic states in children. Our primary objective was to study the clinicoetiological profile of CLDs detected in noncirrhotic stages in children younger than 12 years of age. The secondary objective was to find the hepatic histological correlation of provisional diagnosis by different ranks of doctors.

**Methods:**

This was an observational epidemiological study, cross‐sectional in design, conducted in a tertiary‐care setting over a 2‐year period.

**Results:**

Thirty‐seven cases were enrolled, with a mean ± SD age of 8 ± 4.1 years and a male:female ratio of 1.8:1. Etiologies noted were Wilson disease (*n* = 8), autoimmune hepatitis (*n* = 4), secondary hemochromatosis (*n* = 4), chronic hepatitis B (*n* = 3), chronic hepatitis C (*n* = 2), non‐alcoholic steatohepatitis (*n* = 2), progressive familial intrahepatic cholestasis (*n* = 2), extrahepatic biliary atresia (*n* = 2), Alagille syndrome (*n* = 1), galactosemia (*n* = 1), Gaucher disease (*n* = 1), Niemann‐Pick disease (*n* = 1), and Budd–Chiari syndrome (*n* = 1), with an inconclusive diagnosis in five children. Relevant investigations were ordered more frequently by the specialist consultant (SC) and super specialist (SS) combined in comparison with the senior resident (SR) and junior resident (JR) together. (*P* = 0.0013). Irrelevance of the tests ordered was significantly higher in the junior tier (JR and SR; SR > JR) in contrast to the senior tier of doctors (SC and SS) (*P* < 0.01). The clinicohistological correlation of an etiological diagnosis significantly differed between the junior and senior ranks of physicians. We noted that an ideal clinical acumen could help to avoid liver biopsy for etiological diagnosis in 78.3% (29/37) of the study population.

**Conclusion:**

Interpretation of clinical presentation by the senior set of doctors is preferable, which could obviate the need for liver biopsy regarding diagnosis in a proportion of pediatric CLD patients.

## Graphical abstract

Among the pediatric population, Wilson disease was found to be the most common (21.6%) cause of chronic liver disease diagnosed in noncirrhotic stages. The relevancy of the investigations ordered was more precise (*P* < 0.05) in the senior rank of physicians, whose analytical view should be considered before proceeding with invasive procedures such as liver biopsy in children.

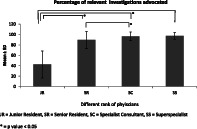

## Introduction

Chronic liver disease (CLD) is a clinicopathological condition that has the potential to progress to end‐stage liver disease (ESLD) if left untreated.^1^ The etiological spectrum of CLD in children includes metabolic, autoimmune, and infective causes. The diagnosis can be merely incidental, as part of a workup of nonrelated or indirectly related symptoms, or is carried out as the children demonstrate various forms of hepatic presentations, often in the later stage of the disease. So, timely diagnosis and treatment before the patient progresses to ESLD is of utmost importance to decrease morbidity and mortality.^2^

Our primary purpose was to find the etiological, clinical, and biochemical spectra and the stage of liver fibrosis in the histology of CLD detected in noncirrhotic stages in children.

As obvious, clinical acumen along with investigational reports allow us to narrow our etiological diagnosis of CLD in pediatric populations. Nevertheless, liver biopsy (LB) remains the gold standard of etiological diagnosis of most of these patients, until and unless the patient has cirrhosis. However, LB is invasive, for which consent from primary caregivers is often difficult to obtain in the pediatric population. LB also has the inherent risk of complications.^3^

Therefore, we secondarily planned to find if a better clinical interpretation of the patient by a higher tier of doctors of related disciplines can obviate the need of LB in children to assess the cause of CLD in children.

## Methods

The study was conducted over a 2‐year period in the Department of Pediatrics in Nil Ratan Sircar Medical College, Kolkata, India, on children younger than 12 years of age, after obtaining the necessary clearance from the Institutional Ethical Committee (IEC) and informed consent from the parents/primary caregivers in‐between September 2017 and August 2019. All patients in Out Patient Department and In Patient Department (OPDs/IPDs) with a prospective diagnosis of CLD were admitted in a particular unit of the Department of Pediatrics, which comprised a junior resident (JR), senior resident (SR), specialist consultant (SC), and super specialist (SS). In addition, based on an institutional decision, intra‐ and interdepartmental pediatric patients with a suspicion of CLD at the level of the consultant were referred to the above pediatric unit. The exclusion criteria for enrollment were presence of hepatic decompensation, pediatric acute liver failure (PALF),^4^ or acute‐on‐chronic liver failure (ACLF)^5^ at any time during the illness duration or course of hospital stay.

On agreement between any two or more of the SR, SC, and SS regarding syndromic diagnosis of CLD, the JR, SR, SC, and SS of the concerned unit were allowed to proceed with further workup of the children separately for the etiology of CLD. Thus, 49 apparently eligible children with a diagnosis of CLD underwent workup.

At the outset of study, JR was a postgraduate (PG) fellow and had just completed 6 months of his training period in the field of Pediatrics. At the same point in time, SR had an experience of 1 year after junior residency, and SC had expertise in working in Pediatrics for more than 5 years after acquiring the required degrees. By the time the study commenced, SS had obtained a postdoctoral degree in Gastroenterology, in addition to being a postgraduate in the concerned specialty.

The present symptomatology, relevant past history, and significant family history were assessed separately by the four different ranks of doctors. The examination details of the children were obtained by the concerned doctors in a segregated manner.

The basic investigations, which were carried out in all children, included complete blood count (CBC), blood for total serum bilirubin – direct (TSB‐D), total serum bilirubin – indirect (TSB‐ID), alanine aminotransferase (ALT), aspartate aminotransferase (AST), alkaline phosphatase (ALP), serum total protein, serum total albumin, international normalized ratio (INR), and ultrasonography of upper abdomen.

Ordering of any of the special investigations was left to the discretion of individual doctors. An internist was entrusted with the task of completing all forms of investigations.

For Wilson disease (WD), pathological values of serum ceruloplasmin and 24‐h urinary copper without and with penicillamine challenge were found to be less than 20 mg/dL and more than 100 and 1600 μg/day, respectively.^6^ The Kayser Fleischer (KF) ring examination was carried out by a slit lamp at the level of consultant of the Department of Ophthalmology. Simplified International Autoimmune Hepatitis Group (IAIHG) criteria with a modification were used to obtain a definite and probable diagnosis of autoimmune hepatitis (AIH).^7^ The modification, whereby patients with positive autoimmune markers were awarded a score of 2—with titers of 1: 40 dilution for antinuclear antibody (ANA) and antismooth muscle antibody (ASMA) and 1:10 for anti‐Liver‐Kidney‐Microsomal (LKM) antibody—and a score of 1 with positivity in less dilution, was required as we were dealing with children.^8^ The method used for the detection of the above markers was Indirect immunofluorescence (IIF).

In addition to the study population, who were referred for high values (>7 kPa)^9^ in transient elastography (TE) (FibroScan, EchoSens, Paris), measurement of liver stiffness for the other children was performed by FibroScan using an S or M probe, wherever the physician believed it necessary, with SS as the operator. The decision to defer this investigation by a maximum of 3 months, wherever applicable, was allowed when patient had presentations of acute hepatitis or cholestasis.^10^

After 10 and 15 days of inpatient evaluation, the provisional diagnosis (PD D10 and PD D15, respectively) of each of the four doctors was recorded separately.

With proper consent, LB was performed by a pathologist with a minimum postresidency experience of more than 3 years using a BardMaxCoreDisposable Core Biopsy Instrument (16GX 16 cm) percutaneously under real‐time image guidance, with strict adherence to prebiopsy preparations and peribiopsy management as per standard guidelines.^11^ LB was postponed to 4 weeks after resolution of symptoms of those children presenting with indicators suggestive of acute hepatitis. For high INR > 1.4, due to cholestasis, administration of an age‐adjusted parenteral injection of vitamin K was carried out before consideration of LB.^12^ After being satisfied regarding the adequacy of the size of the tissue of the biopsy sample, a histological report was prepared by a team of two pathologists, both at the level of consultants, who were unaware of the patient's clinical data. Special stains, if they were to be used, were left to the discretion of the concerned pathologist. Stage of fibrosis was measured using METAVIR staging.^13^ Taking into account clinical details, a histological diagnosis (HD), was made, which was later corroborated with PD D10 and PD D15.

All patients were initially managed symptomatically and, subsequently, were managed specifically as per prevailing protocols of the treatment after diagnosis was established.

Justifiable special investigations were retrospectively analyzed by SC and SS, keeping into mind the clinical points and HD. Thus, the proportions of relevant and irrelevant investigations ordered by the four ranks of doctors were also measured.

### *Statistical analysis*

Data as statistical averages, dispersions, and proportions were compiled in Microsoft Excel ver. 2019 with mean ± SD calculated, and the Chi‐square test and *z* tests were applied as and when applicable at the *P* < 0.05 significance level.

## Results

Thirty‐seven patients were retrospectively enrolled after interpretation of liver histology. In addition, 12 children were excluded as they had histologically proven cirrhosis. A total of 8, 3, and 46 patients with presentations of PALF, ACLF, and hepatic decompensation of CLD, respectively, were also not enrolled. The mean ± SD age of the children was 8 ± 4.1 years with a male:female ratio of 1.8:1. Table 1 shows the breakup of patients according to the etiology with basic demographic parameters, along with predominant mode of presentations.

**Table 1:** The breakup of patients according to etiology with basic demographic parameters along with predominant mode of presentations (*n* = 37)

Etiology	Age (years), mean ± SD	Male:Female	Mode of presentations
Wilson disease (*n* = 8)	9.43 ± 1.46	3:5	Acute hepatitis (*n* = 2)
			Relapsing jaundice (*n* = 2)
			Unexplained hepatomegaly (*n* = 1)
			Unexplained moderate transaminitis (*n* = 1)†
			Detection as part of family screen (*n* = 2)
Autoimmune hepatitis (*n* = 4)	8.96 ± 1.4	1:1	Acute hepatitis (*n* = 1)
			Relapsing jaundice (*n* = 1)
			Unexplained hepatomegaly (*n* = 1)
			Unexplained mild transaminitis (*n* = 1)‡
Secondary hemochromatosis (*n* = 4)	10.6 ± 0.8	4:0	Referred for high TE values in CHA patients
Chronic hepatitis B (*n* = 3)	10 ± 1.95	3:0	Incidental detection (screening program)
Chronic hepatitis C (*n* = 2)	11.5 ± 0.63	2:0	Referred for high TE values in CHA patients
Non‐alcoholic steatohepatitis (*n* = 2)	11.8 ± 2.8	2:0	Referred for high TE values
Progressive familial intrahepatic cholestasis (*n* = 2)	0.75 ± 0.2	1:1	Infantile cholestasis
Extra hepatic biliary atresia (*n* = 2)	0.5 ± 0.07	1:1	Infantile cholestasis
Alagille syndrome (*n* = 1)	0.5	1:0	Infantile cholestasis
Galactossemia (*n* = 1)	0.5	0:1	Infantile cholestasis, recurrent infections
Niemann‐Pick disease (*n* = 1)	0.3	1:0	Infantile cholestasis, FTT, developmental delay
Gaucher disease (*n* = 1)	3.5	1:0	Splenohepatomegaly
Budd‐Chiari syndrome (*n* = 1)	8	1:0	Ascites
Cryptogenic (*n* = 5)	10.6 ± 0.88	3:2	Unexplained hepatomegaly (*n* = 3)
			Unexplained transaminitis (*n* = 2)

† Moderate transaminitis = > 5 < 20 times of ULN.

‡ Mild transaminitis = < 5 times of ULN.

CHA, chronic hemolytic anemia; FTT, failure to thrive; TE, transient elastography.

The most common etiology noted was WD (*n* = 8). Birth from a third‐degree consanguineous marriage was noted in three of the cases. The mean ± SD serum ceruloplasmin and 24‐h urinary copper estimation for these children was 10.13 ± 6 mg/dL and 123 ± 38.7 μg/day, respectively. The KF ring was noted in five children; 24‐h urinary copper estimation after penicillamine challenge was carried out in six patients whose mean ± SD was 1916 ± 332.3 μg/day. Hepatic steatosis was noted in a total of five patients with hepatic histology. As measurement of the dry weight of copper per gram of liver tissue was beyond our scope, we relied on indirect markers such as rhodanine stain in liver histology for our diagnosis, which was positive in five children.

Of the seven patients who underwent workup for infantile cholestasis, two each had progressive intrahepatic cholestasis (PFIC) and extrahepatic biliary atresia (EHBA) with secondary liver injury. The type of PFIC was retrospectively confirmed to be Type 2 on the basis of age of presentation and normal gamma‐glutamyltransferase (GGT) levels. EHBA was diagnosed on the basis of a histological diagnosis coupled with imaging by a pediatric radiologist. The child with Alagille syndrome had the characteristic facies, cardiovascular abnormalities, and vertebral anomalies.

Six children were referred from the Hematology department for high TE values, as part of a separate study protocol. Of them, four children with multitransfused chronic hemolytic anemia (CHA) were diagnosed with SH not amounting to cirrhosis on the basis of iron indices and a classical histology report. The mean ± SD of serum iron (Fe), ferritin, and transferrin saturation (TS) was, respectively, 227 ± 39.1 μg/dL, 2610.3 ± 542.1 ng/mL, and 58 ± 9.1%. The distribution of Fe was panlobular, with periportal to pericentral gradient in three of the patients.

Typical histological features of AIH were present in all the four children. Lymphoplasmacytic infiltrate, interface hepatitis, and emperipolesis were noted in three, two, and one patients, respectively. Two and one patients were positive for the anti‐LKM antibody (1:10 dilution) and ANA (1:40 dilution), respectively. Two patients each fulfilled the IAIHG criteria of definite and probable AIH. Interestingly, one patient presented with high TSB–D values (38.5 mg/dL) and was found to be a case with a protracted course of Hepatitis A virus (HAV)‐induced acute hepatitis in the background of AIH.

Hepatitis B virus (HBV) infection was found to be a part of same family cluster in three children as part of a screening program. Virologic status at baseline, as well as histology, indicated that all patients were in the high‐replicative, low‐inflammatory (HRLI) phase, with liver biopsy noting no fibrosis. Ground‐glass hepatocytes were noted in all of the patients. The mean ± SD of HBV DNA quantification was 10X ^10.3±1.5^ copies/mL.

Two children with CHA were found to be positive for anti‐hepatitis C virus (anti‐HCV) antibody (ELISA method). One of them was adequately chelated and did not fulfill the Fe index criteria of SH. However, the other child demonstrated features of Fe overload histologically, which can occur per se in CHA, as well as in HCV infection. Nevertheless, considering the inhomogeneous hepatic parenchymal distribution of Fe, the final diagnosis was considered to be HCV‐related liver disease. Both the children had detectable RNAs, with one of them being infected with genotype 1 and the other with genotype 3.

Nonalcoholic steatohepatitis (NASH) was the retrospective pathological diagnosis, taking into account anthropometric findings and metabolic workup, in two adolescents who were referred for high TE values from the workup of an allied study. The type of histology was similar in two children, with both having predominant pericentral inflammation and fibrosis.

Intriguingly, one infant at 6 months of age, presenting with intercurrent infections with features of cholestasis, was found to be positive for nonglucose reducing substance (NGRS) based on urine and was subsequently found to be deficient galactose‐1‐phosphate uridyltransferase (GALT) based on blood. The salient histological features noted were “pseudoglandular” transformation with distortion in periportal vasculature.

Unpredictably, one 12–week‐old infant was who presented with cholestasis had liver histological features of marked cholestasis, multinucleated giant cells, and interstitial fibrosis. On the basis of features of failure to thrive (FTT) and developmental delay, skin biopsy with fibroblast culture confirmed the diagnosis of Niemann‐Pick disease Type C at 7 months of age. The diagnosis was missed at all ranks of doctors clinically.

Histology showed anti‐CD68 stain‐positive Gaucher cells, with focal fibrosis and inflammation in a 3.5‐year‐old child presenting with splenohepatomegaly. The diagnosis was otherwise confirmed by estimating glucocerebrosidase enzyme activity in leukocytes.

The patient with Budd–Chiari Syndrome (BCS) was an 8‐year‐old female who presented with high protein (3.2 gm/dL) and high serum ascitic albumin gradient (SAAG) (1.3 gm/dL) ascites for 12 weeks. A Doppler flow study showed thrombus with near occlusion of the inferior vena cava (IVC) and middle hepatic vein (MHV) with a normal splenoportal axis. Hepatic histology, after stabilization of the girl, revealed necrosis, congestion, and mild fibrosis (F1). Surprisingly, however, prothrombotic workup was negative; with no gastric or esophageal varices noted by the gastroscope (Olympus GIF‐XP180N, Pediatric Endoscope, OD; 5.5 mm).

The five children whose diagnosis could not be established even after histological interpretation were labeled as having cryptogenic CLD. Three of them presented with unexplained hepatosplenomegaly and the other two with transaminitis. One of these children with ASMA serology positivity (1:40 dilution) could not meet the probable AIH score on the basis of modified simplified IAIHG group criteria with regard to the retrospective analysis of data. Another patient had low serum ceruloplasmin (15 mg/dL) with an above‐normal range of 24‐h urinary copper (62 μg/day) without penicillamine challenge. However, the diagnosis was not established as histology was not compatible.

Table 2 shows the basic laboratory parameters, including stage of fibrosis, in the histology of the case series.

**Table 2:** The basic laboratory parameters, including grade of fibrosis, in histology of the case series

Etiology	TSB‐D	TSB‐Indirect	ALT	AST	ALP	Serum Pr	S alb	INR	TE (kPa)	Fibrosis †			
	(mg/dL)	(mg/dL)	(IU/L)	(IU/L)	(IU/L)	(g/dL)	(g/dL)			F0	F1	F2	F3
	Mean ± SD	Mean ± SD	Mean ± SD	Mean ± SD	Mean ± SD	Mean ± SD	Mean ± SD	Mean ± SD	Mean ± SD	*n*(%)	*n* (%)	*n* (%)	*n* (%)
Wilson disease (*n* = 8)	4.21 ± 3.95	1.06 ± 0.88	291.75 ± 249.07	278.25 ± 242.29	247.63 ± 39.42	7.19 ± 0.34	3.93 ± 0.31	1.29 ± 0.17	8.22 ± 1.67	3 (37.5)	3 (37.5)	1 (12.5)	1 (12.5)
Autoimmune hepatitis (*n* = 4)	10.2 ± 16.62	1.97 ± 2.45	477.25 ± 378.20	478.5 ± 414.03	308.25 ± 158	7.1 ± 0.37	3.7 ± 0.38	1.25 ± 0.12	8.85 ± 1.57	1 (25)	2 (50)	1 (25)	—
Secondary hemochromatosis (*n* = 4)	1.12 ± 0.38	4.1 ± 1.06	121 ± 61.5	112.5 ± 61.08	288.75 ± 17.96	6.35 ± 0.34	3.12 ± 0.09	1 ± 0.19	10.05 ± 0.5		1 (25)	2 (50)	1 (25)
Chronic hepatitis B (*n* = 3)	1.2 ± 0.2	0.4 ± 0.1	44 ± 4	44.66 ± 6.42	267 ± 53	7.26 ± 0.20	4 ± 0.2	1.05 ± 0.07	5.36 ± 1.26	3 (100)	—	—	—
Chronic hepatitis C (*n* = 2)	1.4 ± 0.56	4.45 ± 0.91	105 ± 24.04	78 ± 22.62	289 ± 18.38	6.4 ± 0.70	2.95 ± 0.07	1.25 ± 0.07	11.6 ± 1.97	1 (50)	1 (50)	—	—
Non‐alcoholic steatohepatitis (*n* = 2)	1.05 ± 0.21	0.45 ± 0.07	75 ± 9.89	87 ± 12.72	306 ± 55.15	7.5 ± 0.14	3.9 ± 0.14	1 ± 0.14	9.9 ± 0.14	—	1 (50)	1 (50)	—
Progressive familial intrahepatic Cholestasis (*n* = 2)	10.7 ± 2.68	1.8 ± 0.28	68 ± 5.65	60 ± 8.48	485 ± 52	7.1 ± 0.14	3.7 ± 0.14	1.55 ± 0.07	‡	—	2 (100)	0	—
Extra hepatic biliary atresia (*n* = 2)	13.6 ± 3.95	1.5 ± 0.42	94 ± 11.31	98 ± 48.08	546 ± 84.85	6.45 ± 0.63	3.45 ± 0.07	1.45 ± 0.07	‡				
Alagille syndrome (*n* = 1)	10	3	78	58	604	7	3.6	1.7	‡		—	2 (100)	—
Galactossemia (*n* = 1)	13	2.4	156	158	288	6	3	1.5	‡				
Niemann‐Pick disease (*n* = 1)	14	2.6	124	102	256	6.2	3.2	1.5	‡	1 (100)	—	—	—
Gaucher disease (*n* = 1)	2.8	0.6	202	256	324	6.4	3.4	1.1	‡	—	1 (100)	—	—
Budd‐Chiari syndrome (*n* = 1)	5.6	1.4	344	188	254	6.8	3.2	1.2	‡	—	1 (100)	—	—
Cryptogenic (*n* = 5)	2.65 ± 0.3	0.9 ± 0.22	132.2 ± 59.69	128 ± 49.49	171.8 ± 35.18	6.58 ± 0.38	3.44 ± 0.35	1.12 ± 0.22	10.72 ± 0.87	—	1 (20)	3 (60)	1 (20)
Total (*n* = 37)	5.10 ± 6.79	1.75 ± 1.54	192.1 ± 206.37	183.02 ± 209.2	297.48 ± 118.5	6.81 ± 0.52	3.57 ± 0.41	1.24 ± 0.22	9 ± 2.13	9 (24.3%)	15 (40.5)	10 (27%)	3 (08.1%)

^†^ Fibrosis according to METAVIR staging.

^‡^ TE values not taken into consideration where TSB and ALT, AST continued to be high (>5 mg/dL and > 5 ULN, respectively) in spite of optimal treatment after 12 weeks of therapy.

ALP, alkaline Phosphatase; ALT, alanine aminotransferase; AST, aspartate aminotransferase; INR, international normalized ratio; TSB‐D, total serum bilirubin – direct; TSB‐ID, total serum bilirubin – indirect; S alb, serum total albumin; Serum Pr, serum total protein.

**Figure 1:**
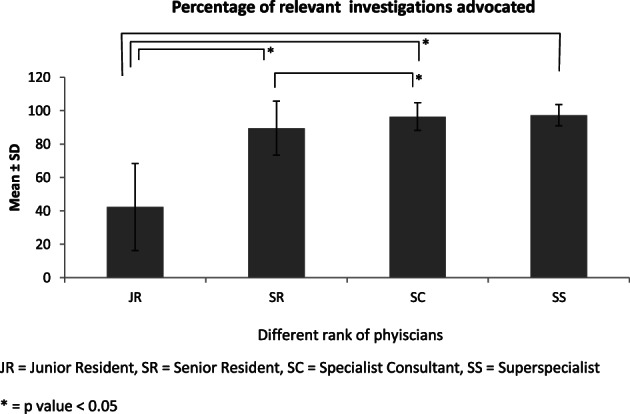
The mean ± SD of the percentage of relevant tests ordered by the designated physicians. *P value <0.05. JR, junior resident; SC, specialist consultant; SR, senior resident; SS, super specialist. (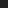), mean.

**Figure 2:**
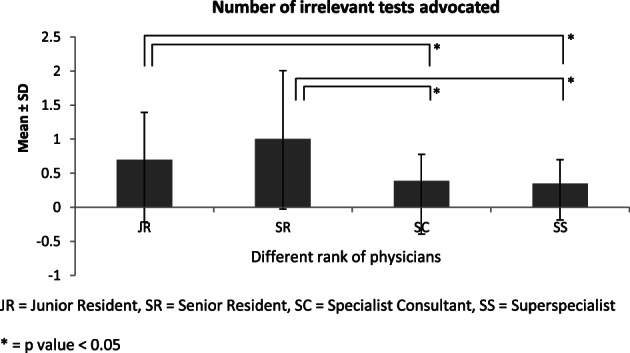
The mean ± SD of the number of irrelevant tests ordered by the designated physicians. *P value <0.05. JR, junior resident; SC, specialist consultant; SR, senior resident; SS, super specialist. (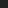), mean.

Figure 1 shows the mean ± SD of the percentage of relevant tests advised by JR, SR, SC, and SS. When relevant investigations ordered were compared between the subsequent higher tier of doctors, using the *z* test, statistical significance was noted between JR and SR (*P* < 0.001) and SR and SC (*P* < 0.004) but not between SC and SS. (*P* = 0.32). Figure 2 shows the mean ± SD of the number of irrelevant tests ordered by four ranks of doctors, which interestingly demonstrates that SR was more overenthusiastic while investigating compared to JR. The statistical importance of the irrelevant tests planned was significant only between JR and SC (*P* = 0.019), JR and SS (*P* < 0.001), SR and SC (*P* < 0.001), and SR and SS (*P* < 0.001).

Table 3 shows the proportion of correctness of PD D10 and PD D15 by the designated doctors of the 32 children, where a definite final diagnosis could be reached.

**Table 3:** The proportion of correctness of PD D10 and PD D15 by the designated doctors of the 32 children for whom a definite final diagnosis could be reached

	Provisional diagnosis				*P* value
	Correct		Incorrect		
Rank of doctors	*n*	%	*n*	%	
Day 10					
JR	15	46.88	17	53.13	Chi‐square = 13.9, df = 9, *P* = 0.003
SR	24	75	8	25	
SC	27	84.38	5	15.63	
SS	26	81.25	6	18.75	
Day 15					
JR	16	50	16	50	Chi‐square with Yates correction = 20.84, df = 9, *P* < 0.001
SR	26	81.25	6	18.75	
SC	29	90.63	3	9.38	
SS	29	90.63	3	9.38	

JR, junior resident; PD D10, provisional diagnosis at D10 of admission; PD D15, provisional diagnosis at D15 of admission; SC, specialist consultant; SR, senior resident, SS, super specialist.

## Discussion

Our study is one of the few studies that focused on the spectrum of the subset of noncirrhotic CLD in a pediatric population in a tertiary‐care center. Dhole *et al*. noted liver histology in CLD in children; however, that case series included cirrhotic patients, which makes it distinctly different from our study design.^1^

The results highlight that WD is a frequent cause of CLD in our population, which has been previously reported by the authors.^14^ Serum ceruloplasmin was higher than normal in 12.5%, and the urinary copper was lower than 100 μg/day in 37.5% of patients, which is comparable to what has been reported in the literature.^15^ The KF ring was noted in a higher proportion of patients (62.5%) than previously cited by the authors themselves for WD.^16^ For WD, the Leipzig score could not be calculated in our cases as the dry weight of Cu estimation was beyond our scope.^17^

The proportion of AIH as etiology in our case series of CLD is higher in contrast to what has been reported in the literature on adult populations as diseases like AIH, with a faster rate of progression, will obviously manifest more frequently in the younger age group.^18^

All the four children with SH had high elastography values (Mean ± SD, 10 ± 0.5 kPa) and significant fibrosis (≥F2) and are adolescents with CHA, with poor chelation. A literature search revealed that Prati *et al*. documented a single‐center experience of 117 cases of thalassemic patients in this regard; however, most of their study population was HCV positive (91%).^19^

Contrary to the contrasting natural history of HBV and HCV, the patients with HCV had more fibrosis compared to those with HBV. This may be explained by the fact that the HCV‐infected children are multitransfused CHA patients with an additional secondary Fe overload.^20^, ^21^, ^22^

With changing socioeconomic scenarios; it is not natural that the incidence of NASH is on the rise. We, however, failed to demonstrate portal predominant NASH as evident in the literature.^23^

Among the patients with infantile cholestasis, PFIC and its type were only established after clinical and biochemical correlation with the histopathological examination (HPE) report.^24^ EHBA children had significant fibrosis (≥F2), which is compatible with its natural history.^25^

The patient with galactosemia presented unusually late; and diagnosis was thus clinically overlooked by the junior tier of doctors. The histological features were, however, similar to what are documented.^26^ Even though pediatric case series of BCS have been documented across continents, yet reports of children with hepatic presentations of Niemaan‐Pick Disease are quite scant from Asia‐Pacific region.^27^, ^28^, ^29^

Our study obviously reflects the common causes encountered in etiologies of CLD; however, this may not be an ideal representation of the proportion of overall cases of CLD as we had left out cirrhotic patients, both compensated and decompensated. In continuation, we did not extend our scope to analyzing the correlation between TE values and stage of fibrosis in the pediatric population across various etiologies, which is yet to be established precisely.^30^ Moreover, we were unable to compare our objective findings of agreement of relevant and irrelevant tests ordered in the different tiers of physicians as, probably for the very first time, we quantified the diagnostic acumen of the varied ranks of medical professionals.

As previously mentioned, the planning for investigations was more precise in the senior ranks of doctors. Analyzing the data, we also found that a better clinical analysis helped avoid liver biopsy for etiological diagnosis in as many as 29 children (78.3%). Therefore, we suggest that the clinical interpretation of data of patients by the senior rank of doctors and their advocacy should be part of the protocol before planning for an LB in children. Observations from further multicentric studies are needed to compare our findings.
